# Preservatives and Food

**Published:** 1924-11

**Authors:** 


### PRESERVATIVES IN FOOD.

The Departmental Committee of the Ministry of Health which has been inquiring into the use of preservatives and colouring matter in food has now presented its final report. Its recommendations are simple and drastic. The Committee are unanimously of opinion that "the use of preservatives should be prohibited in all articles of food and drink offered or exposed for sale, whether manufactured in this country or imported, except in a few definite and specific cases." The list of exceptions is short, and the only preservatives to be permitted are benzoic and sulphurous acids and their salts, as being the least harmful. If these recommendations are carried into effect we shall at last be able to count upon eating food in England as carefully protected as it is in America At present preservatives, however unnecessary and however harmful, appear to be used at haphazard. The Committee divide them into three groups as worst, not quite so bad, and least harmful. In the blackest list they place formaldehyde and its derivatives and the fluorides; in the next boric acid, salicylic acid, and their salts; the least harmful are those which they suggest should be permitted in "a few definite and specific cases." It is essential to remember that the object of adding preservatives to food is not merely to prevent or delay fermentation or putrefaction, but to mask those unhealthy, and often deadly, conditions when they exist.

The extent to which we eat and drink these preservatives is luridly suggested by some of the discoveries of the Committee. Thus, as many as 175 grains of boric acid have been found in a pound of margarine, 140 in butter, and 110 in bacon. Cream, imported liquid eggs, sausages, potted meat and fish and some beverages are all "doctored." In face of these facts the Committee find it "easy to imagine a reasonable meal which might contain twenty or even more grains of boric acid, besides other preservatives." We observe with satisfaction that, in addition to these highly necessary condemnations, the Committee go farther and attack some of the causes which have led to the widespread use of preservatives, the most innocent of which are, at the best, unfavourable to the human organism. Thus, they emphasise the deficiency in this country of means of transport suitable for foodstuffs as well as the lack of facilities for refrigeration. We have hardly learned the elements of the use of ice, which, even in the hottest weather, is almost unknown outside London and the greatest towns.

Valuable as this report is, we shall be no whit better off if its recommendations are allowed to remain a dead letter. Public opinion will support any Government which gives effect to them. It would, indeed, go farther and support much more drastic measures than have yet been taken for stopping that adulteration of food upon which we print this month an important article by Dr. Hutt, the Medical Officer of the Borough of Holborn. In this respect we are woefully behind not only America, but some of our own colonies.

